# Supplementary material for: Genome-Wide Association Study Reveals CLEC7A and PROM1 as Potential Regulators of Paracoccidioides brasiliensis-Induction of Cytokine Production in Peripheral Blood Mononuclear Cells
Source: J Fungi (Basel). 2023 Mar 30;9(4):428. doi: 10.3390/jof9040428 (PMC10144159; doi:10.3390/jof9040428)
Supplement: Supplementary file 1 [file jof-09-00428-s001.zip › jof-2177805-supplementary.pdf]

Supplementary Information

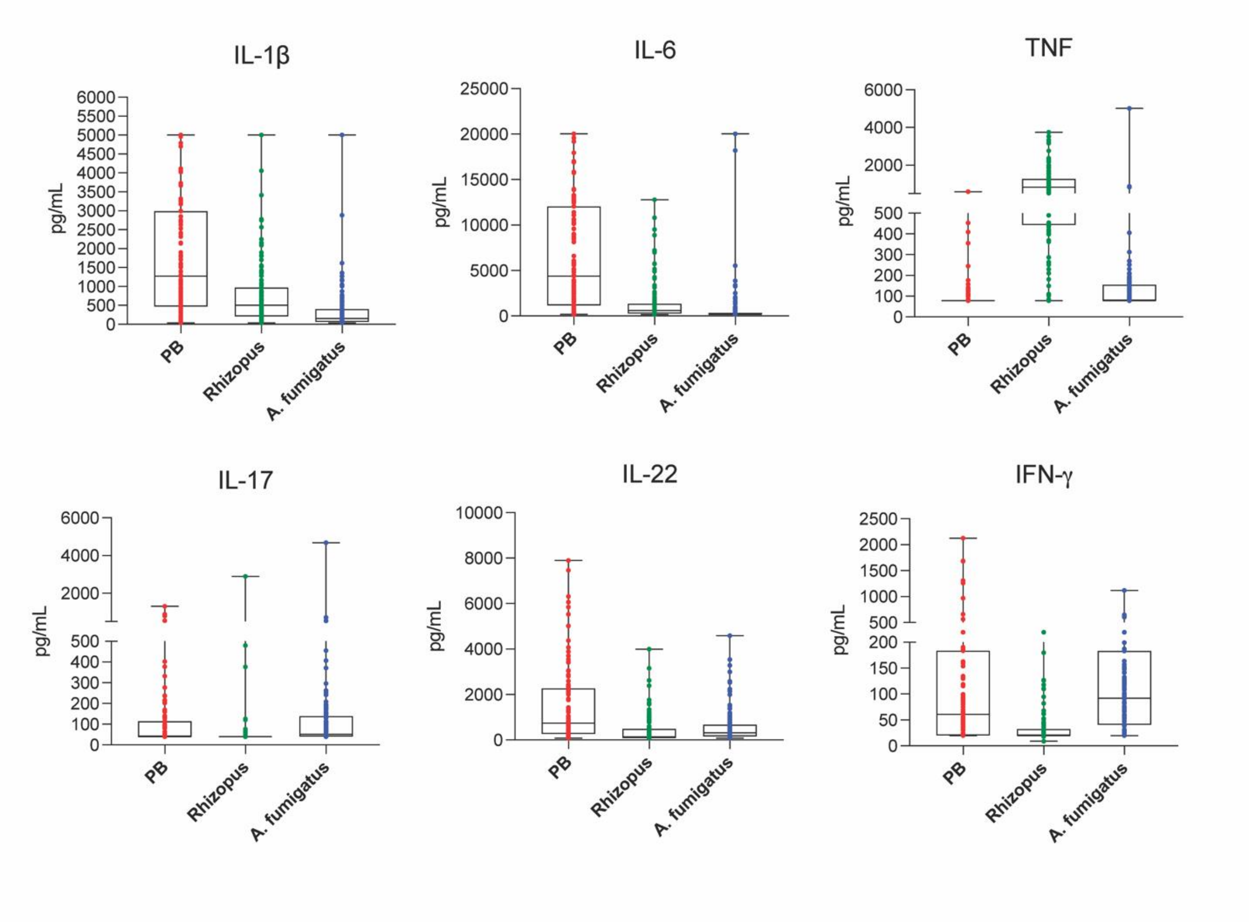

Supplementary Figure S1: Inter-individual variation upon stimulation. IL-1β, TNF, IL-6 (24 h of incubation), IFNγ, IL-22 and IL-17 (seven days of incubation) levels after PBMC stimulation with heat-killed yeasts of *P. brasiliensis,* conidia of *A. fumigatus* and or *R. oryzae* (2.5 PBMCs:1 fungus; cytokines were measured by ELISA in pg/mL); The data is displayed as box-plots with median, quartiles with maximal and minimal values.

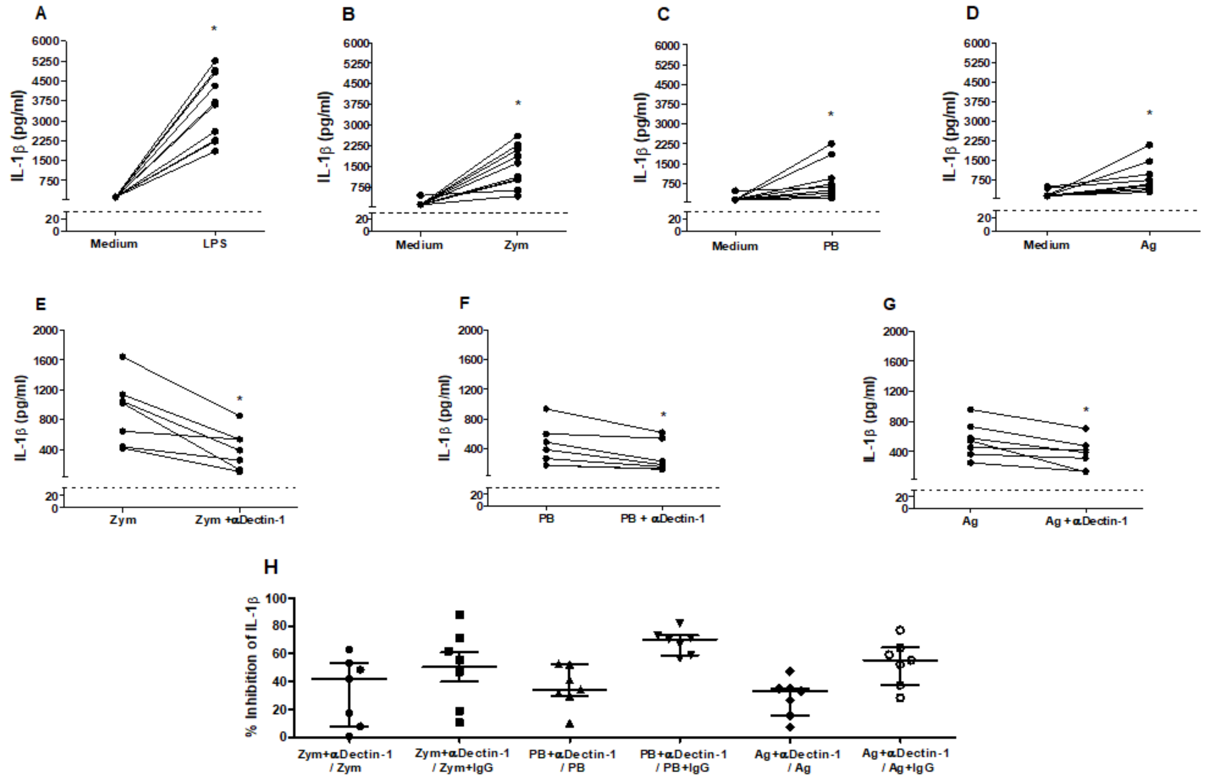

Supplementary Figure S2. **Blocking of Dectin-1 decreases IL-1β production induced by *P. brasiliensis*.** PBMCs (2.5 × 10^5 cells/well) obtained from healthy donors were incubated in the absence or presence of neutralizing anti-Dectin-1 (αDectin-1 at 10 μg/mL) or control IgG antibodies (IgG at 10 μg/mL) for 1 h. Then, the cells were stimulated with 100 ng/mL of lipopolysaccharide (LPS), 50 μg/mL of Zymosan (Zym), 1 x 10^6 yeasts/mL (2.5 PBMCs:1 yeast) of heat-killed *P. brasiliensis* (PB) or 20 μg/mL of particulate antigens from *P. brasiliensis* yeasts (Ag) for 24 h. The supernatant was collected to measure the amount of IL-1β by ELISA. In A-D: The IL-1β production induced by LPS, Zym, PB or Ag compared to the negative control, Medium (n = 10). In E-G: The amount of IL-1β produced after stimulation of PBMCs with Zym, PB or Ag, in the absence or presence of neutralizing anti-Dectin-1 antibodies (n = 7) (Wilcoxon paired test, * p <0.05). (H) Percentage of inhibition of IL-1β due to blocking of Dectin-1 in relation of stimuli in the absence or presence of IgG control antibodies (n = 7). The data are presented as individual values, medians and interquartile ranges.

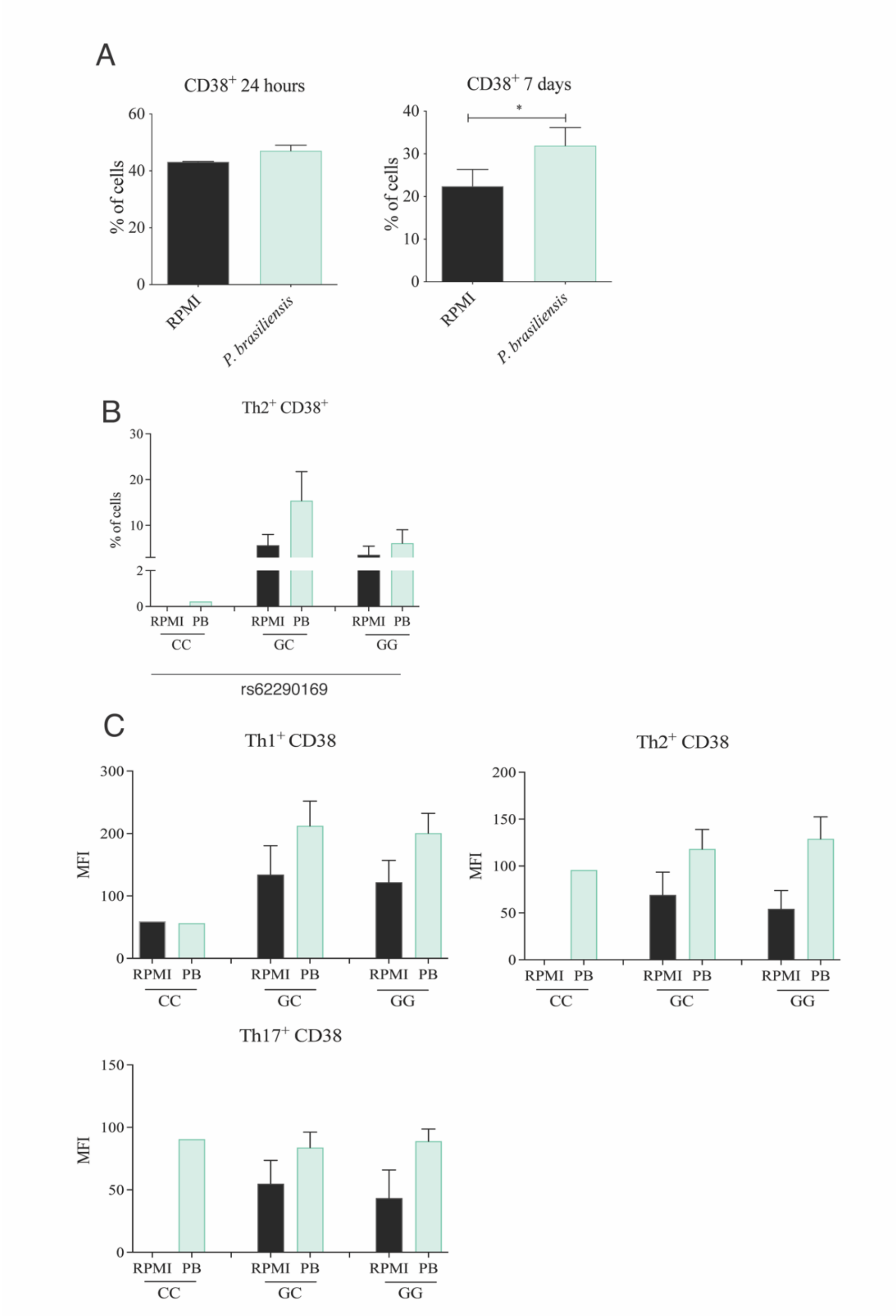

Supplementary Figure S3. **Frequency of CD38 expressing cells.** (A) Frequency of cells expressing CD38 in total PBMCs stimulated with *P. brasiliensis* for 24 hours and 7 days, by flow cytometry (n = 6 ,Wilcoxon paired test, * $p < 0.05$). (B) CD38 protein expression upon exposure to *P. brasiliensis* for seven days assessed by flow cytometry in Th2 cells of individuals part of the 200FG cohort carrying the different genotypes of the rs62290169 SNP (*PROM1* gene) CC (n = 1), GC (n = 4), GG (n = 6) and (D) CD38 mean fluorescence intensity (MFI) in Th1, Th2 and Th17 cells (Wilcoxon paired test, * $p < 0.05$).

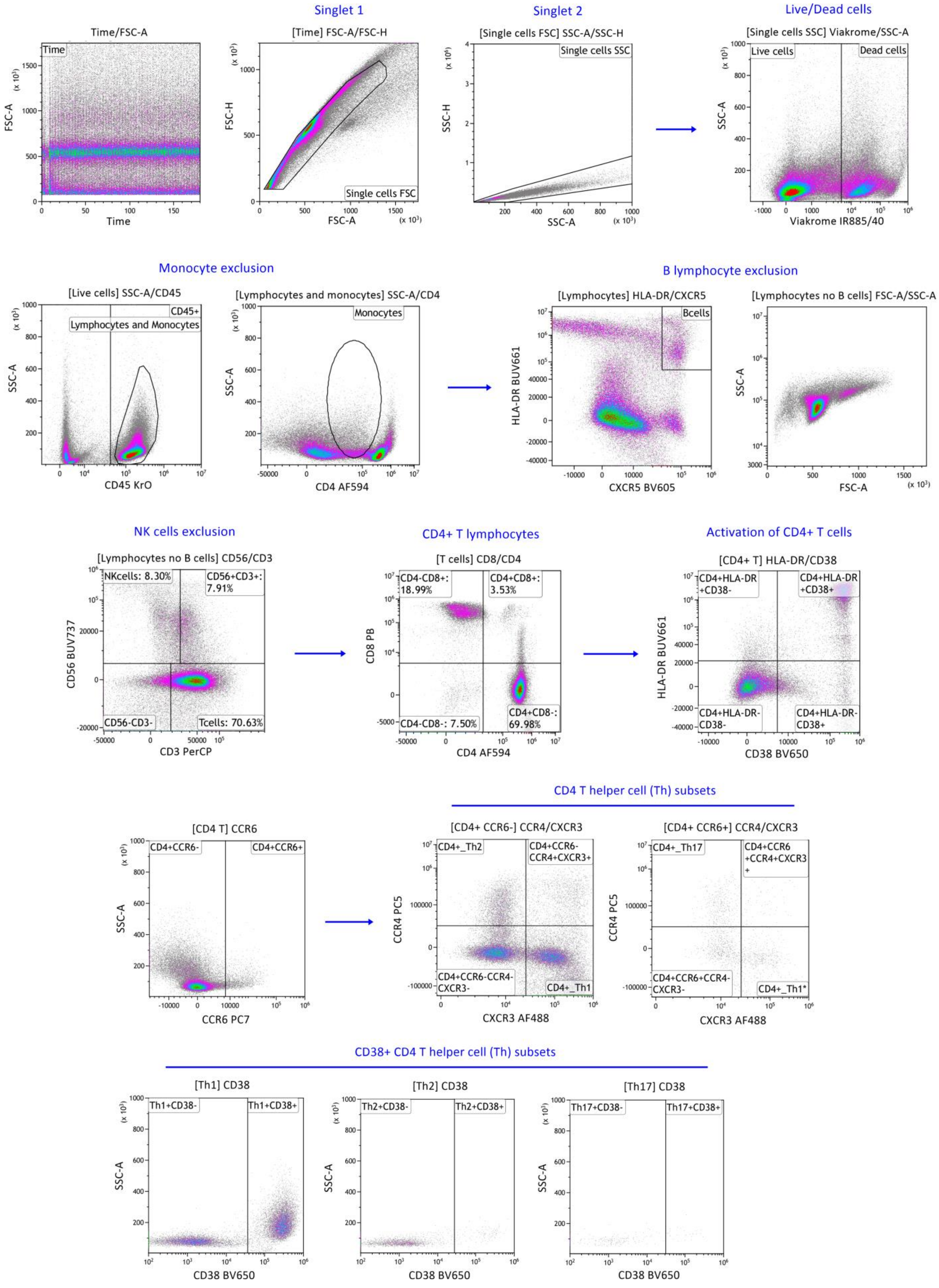

Supplementary Figure S4. **Representative dot plots of the indicated receptors in PBMCs upon *P. brasiliensis* exposure for 7 days.** Related to figure 4 and supplementary 2. The cells were

stained 4

with anti-HLA-DR, anti-CD56, anti-CD3, anti-CD4, anti-CD8, anti-CD45, anti-CXCR5, anti-CD38, anti-CXCR3, anti-CCR4, anti-CCR6 and ViaKrome as viability dye. Percentages of Th1, Th2, and Th17 expressing CD38 were obtained from the indicated $CD4^+$ T helper cell subsets.
